# Supplementary figures and images for: Phosphoregulation accommodates Type III secretion and assembly of a tether of ER-Chlamydia inclusion membrane contact sites
Source: eLife. 2022 Jul 15;11:e74535. doi: 10.7554/eLife.74535 (PMC9286742; doi:10.7554/eLife.74535)

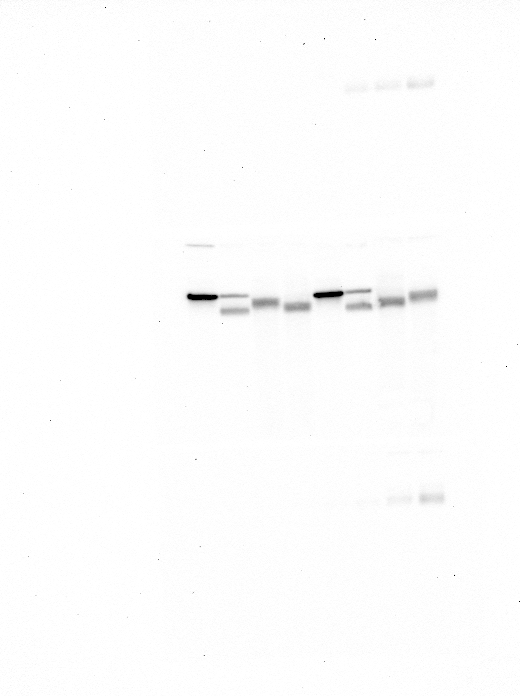

Supplement: Figure 1—source data 4. [file elife-74535-fig1-data4.tif]

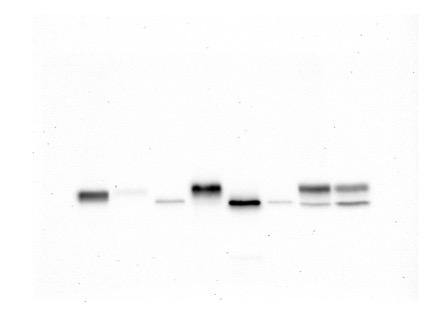

Supplement: Figure 1—source data 5. [file elife-74535-fig1-data5.tif]

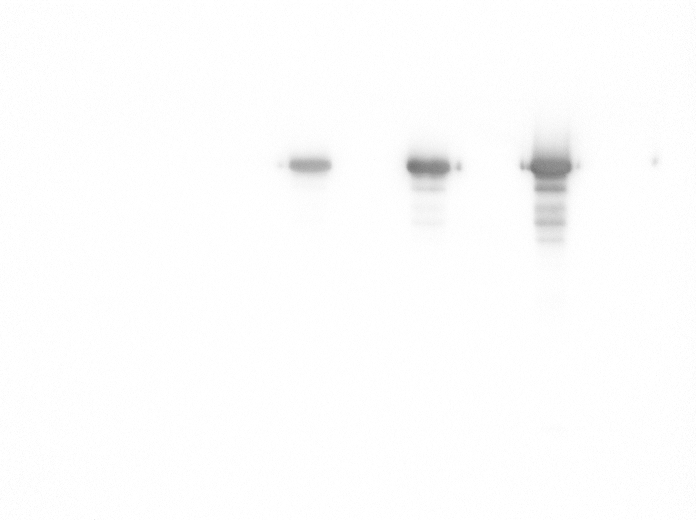

Supplement: Figure 1—source data 6. [file elife-74535-fig1-data6.tif]

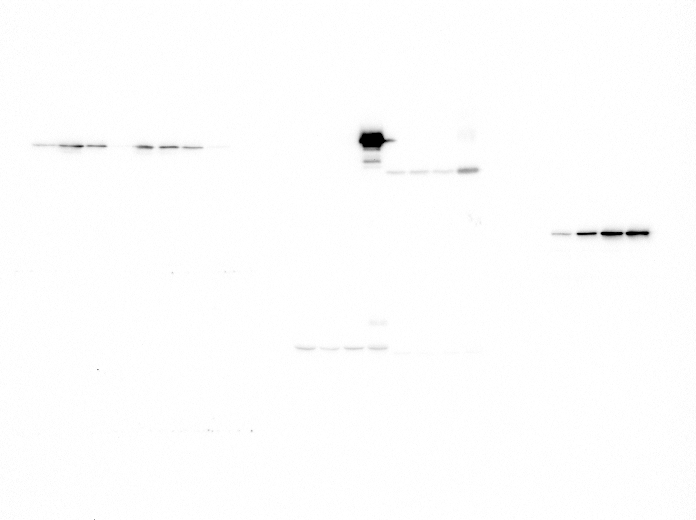

Supplement: Figure 1—source data 7. [file elife-74535-fig1-data7.tif]

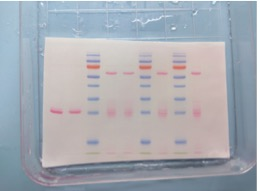

Supplement: Figure 1—source data 8. [file elife-74535-fig1-data8.tif]

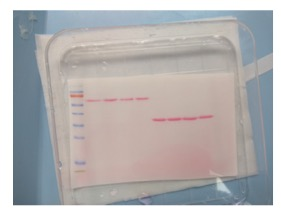

Supplement: Figure 1—source data 9. [file elife-74535-fig1-data9.tif]

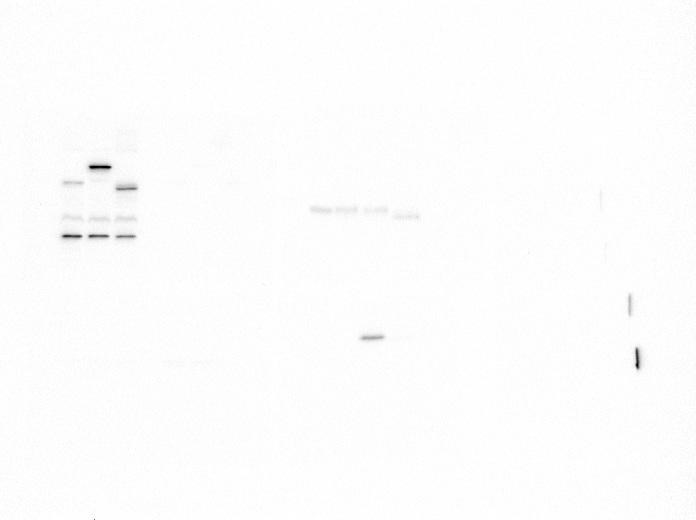

Supplement: Figure 2—source data 4. [file elife-74535-fig2-data4.tif]

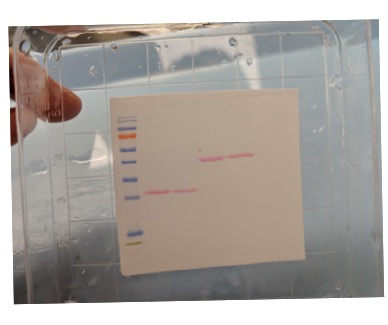

Supplement: Figure 2—source data 5. [file elife-74535-fig2-data5.tif]

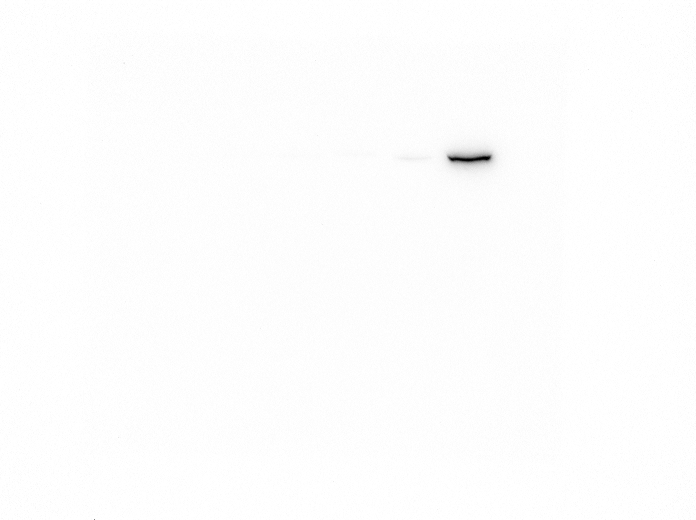

Supplement: Figure 2—source data 6. [file elife-74535-fig2-data6.tif]

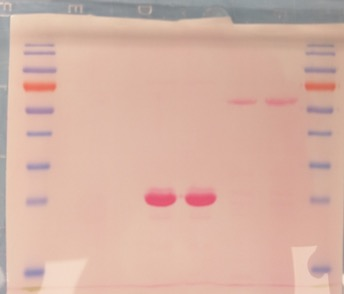

Supplement: Figure 2—source data 7. [file elife-74535-fig2-data7.tif]

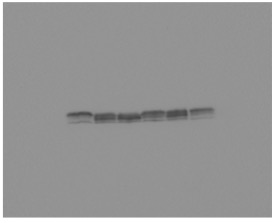

Supplement: Figure 3—source data 4. [file elife-74535-fig3-data4.tif]

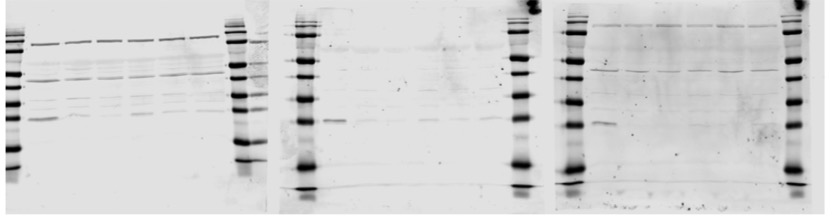

Supplement: Figure 3—source data 5. [file elife-74535-fig3-data5.tif]

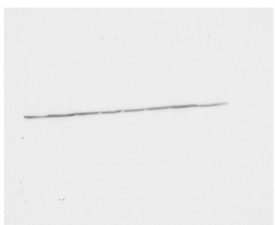

Supplement: Figure 3—source data 6. [file elife-74535-fig3-data6.tif]

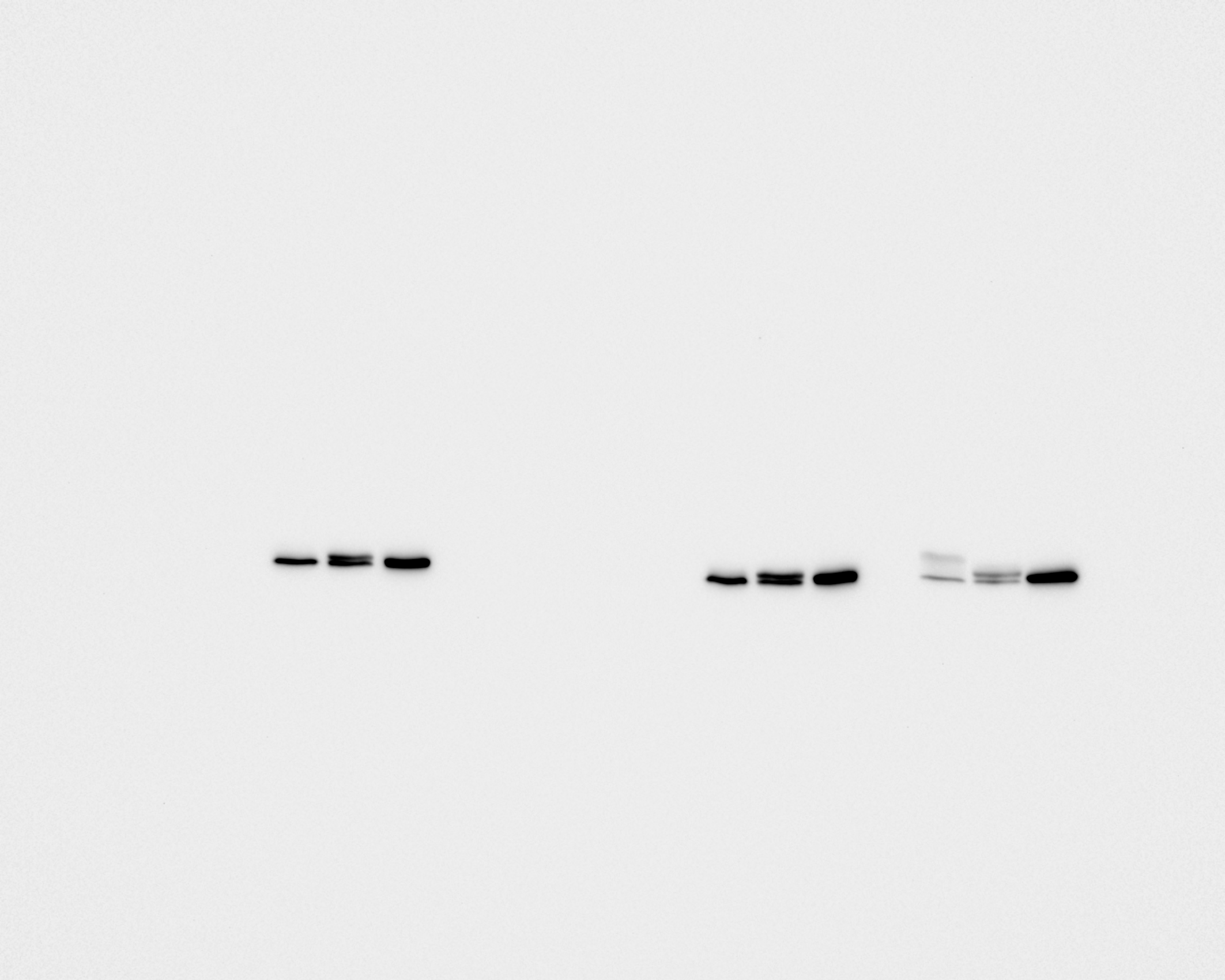

Supplement: Figure 3—source data 7. [file elife-74535-fig3-data7.tif]

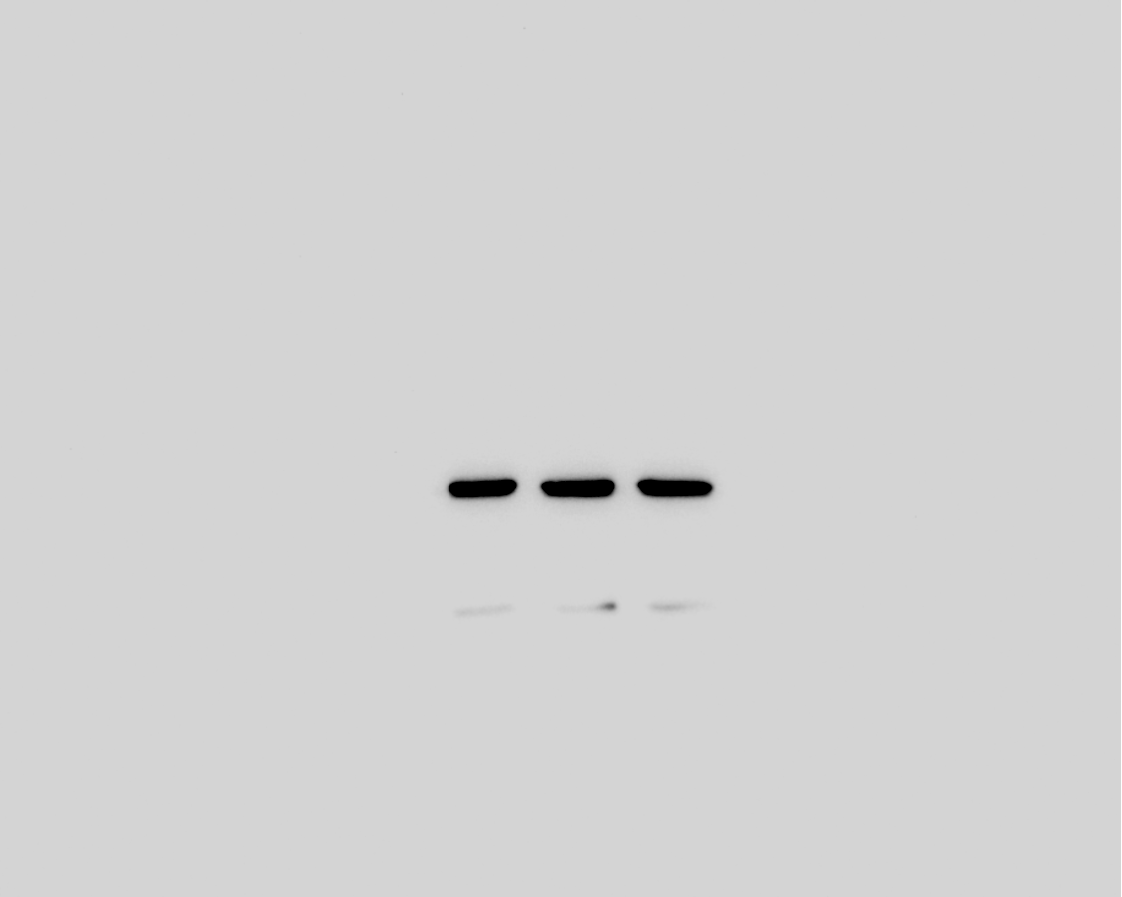

Supplement: Figure 3—source data 8. [file elife-74535-fig3-data8.tif]

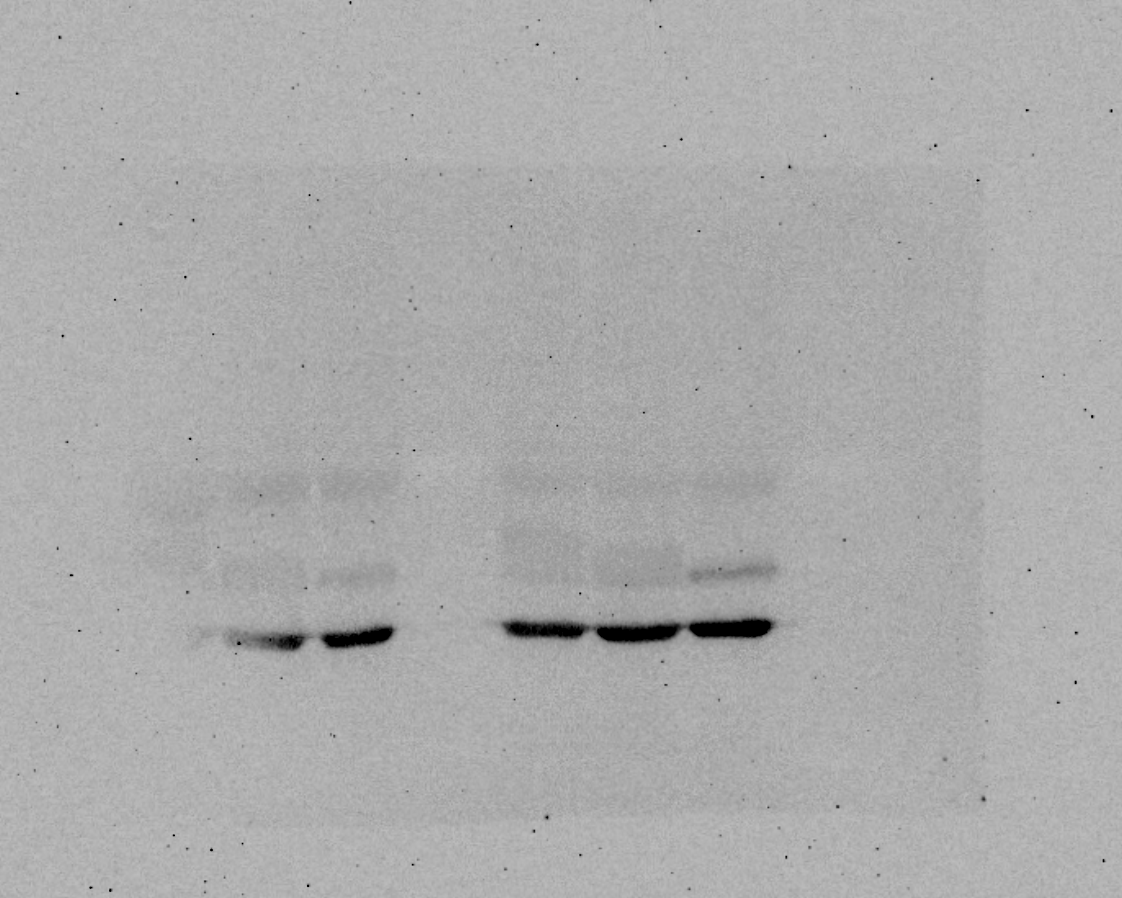

Supplement: Figure 3—source data 9. [file elife-74535-fig3-data9.tif]

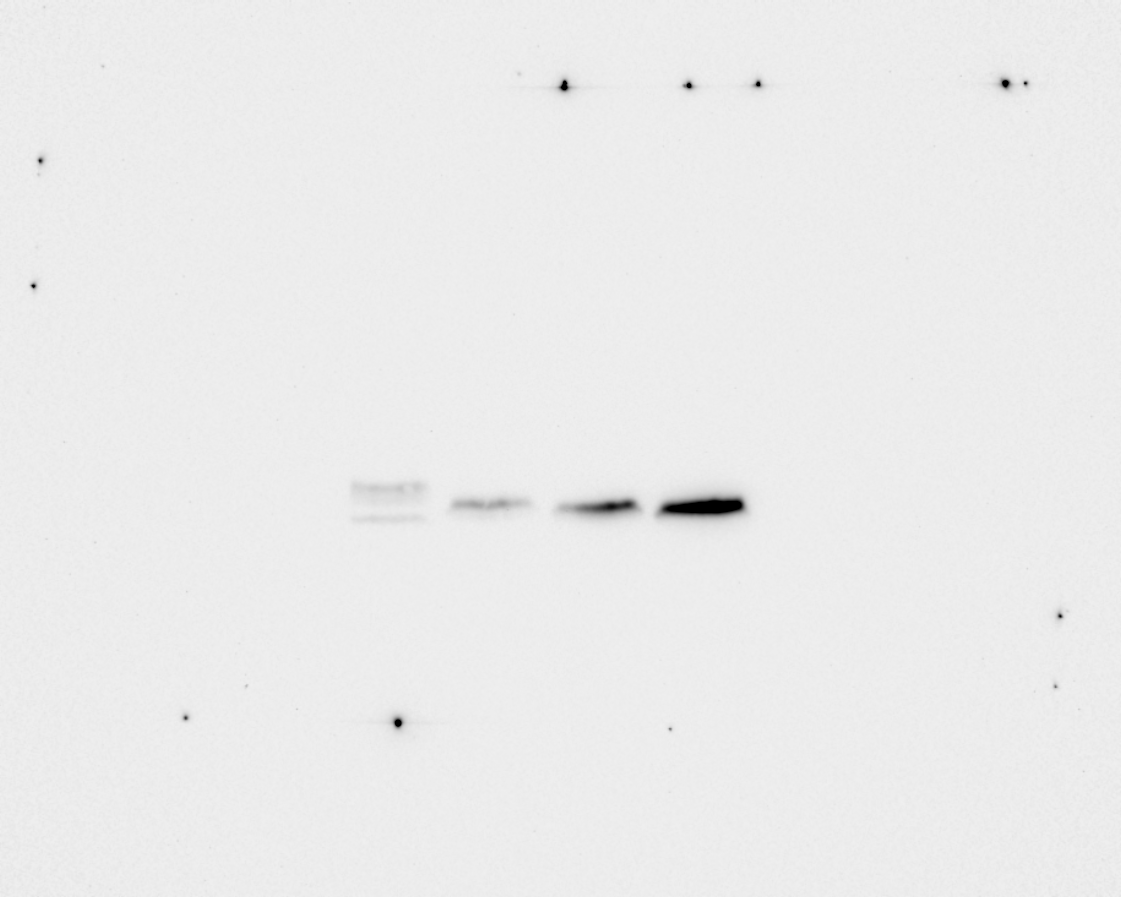

Supplement: Figure 3—figure supplement 3—source data 2. [file elife-74535-fig3-figsupp3-data2.tif]

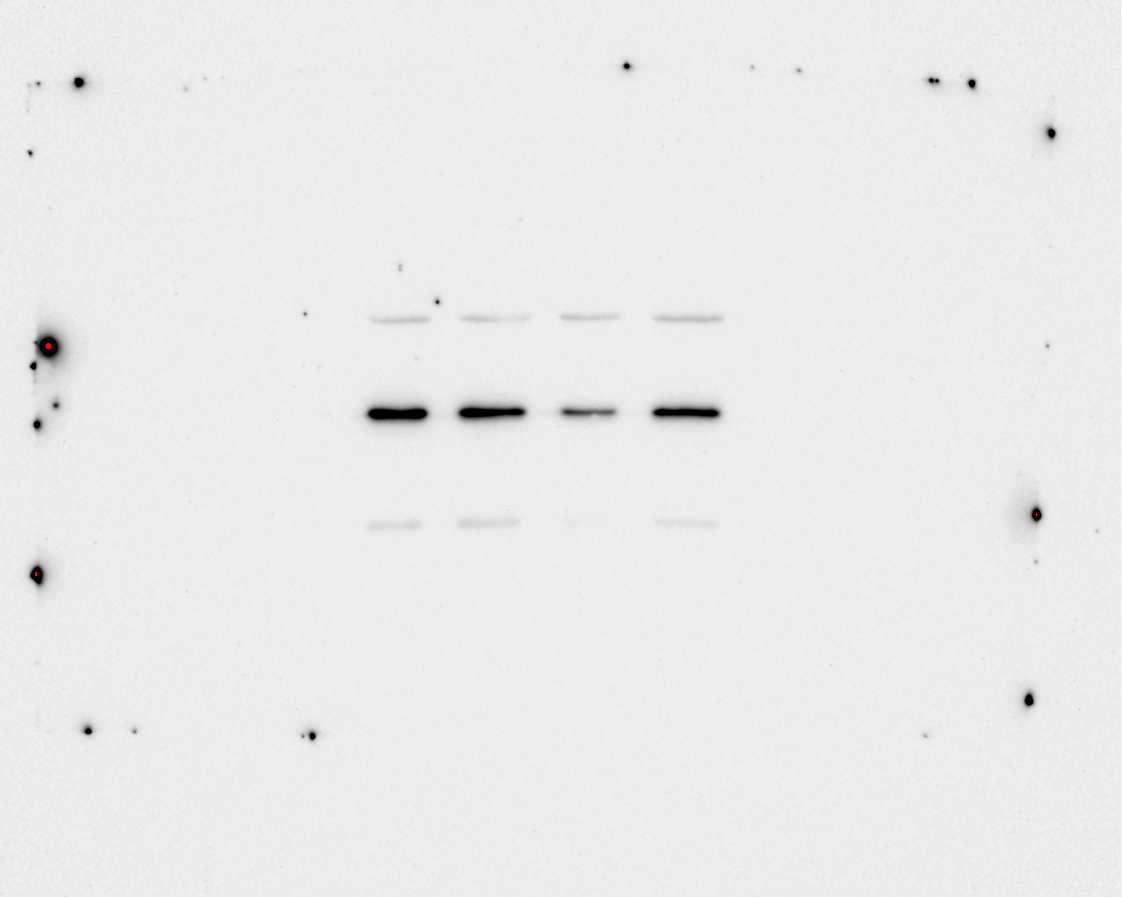

Supplement: Figure 3—figure supplement 3—source data 3. [file elife-74535-fig3-figsupp3-data3.tif]

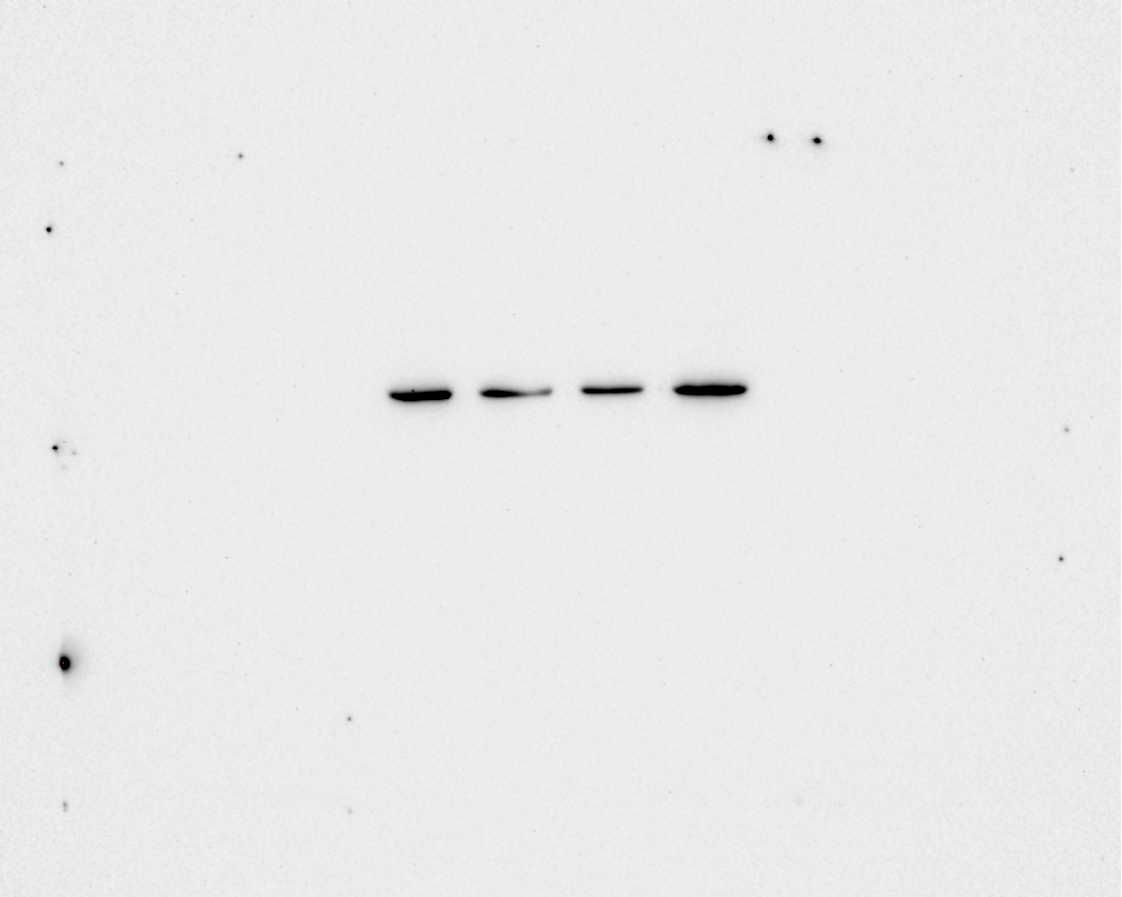

Supplement: Figure 3—figure supplement 3—source data 4. [file elife-74535-fig3-figsupp3-data4.tif]

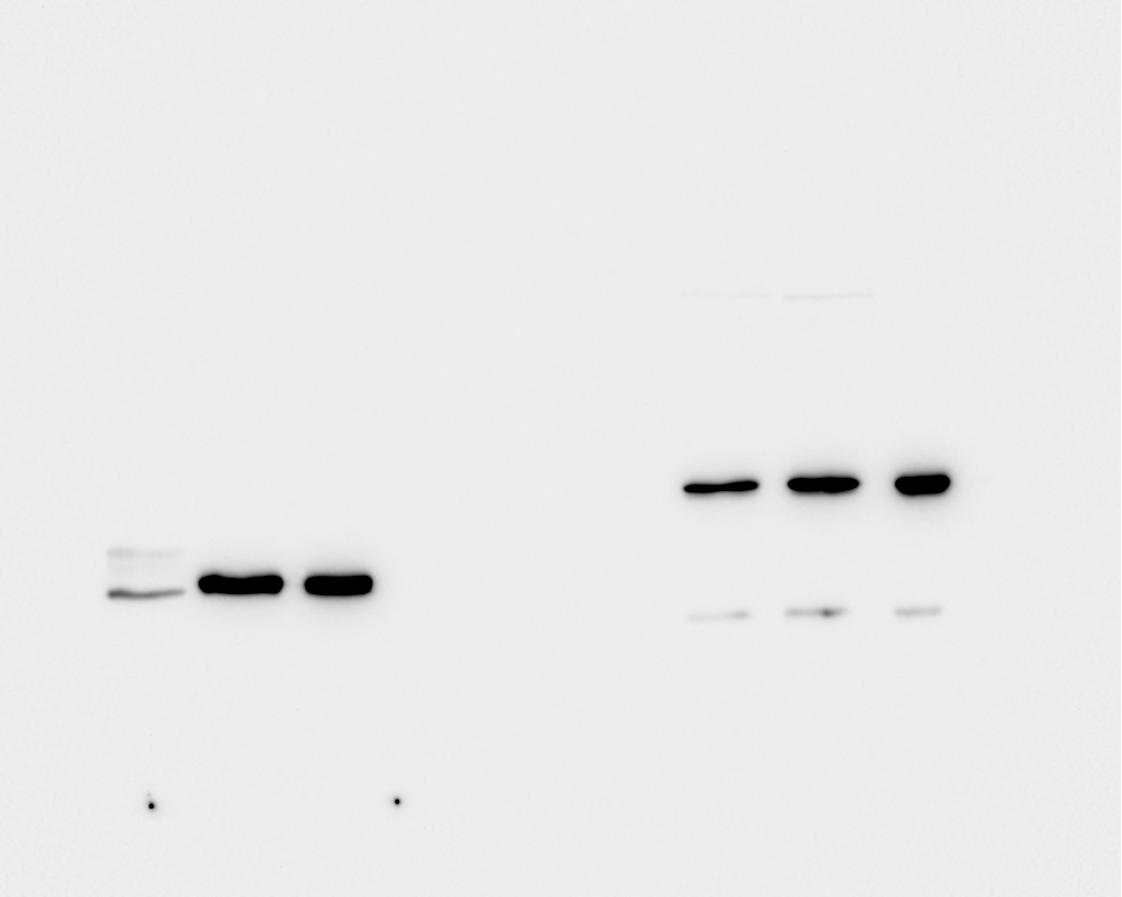

Supplement: Figure 5—source data 3. [file elife-74535-fig5-data3.tif]

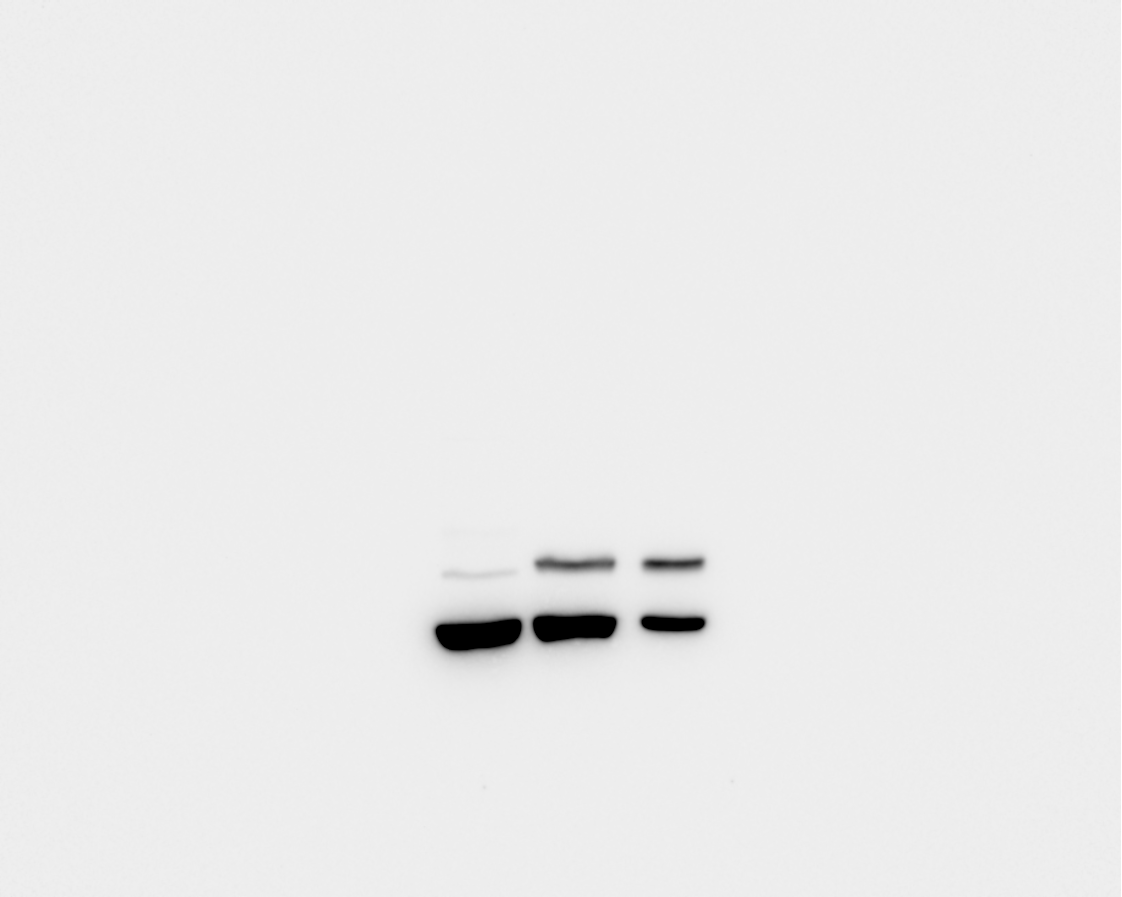

Supplement: Figure 5—source data 4. [file elife-74535-fig5-data4.tif]

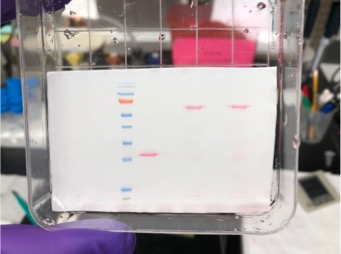

Supplement: Figure 5—figure supplement 2—source data 1. [file elife-74535-fig5-figsupp2-data1.zip › CK2-IncV_eLife3_Fig5S4_SourceData4.tif]

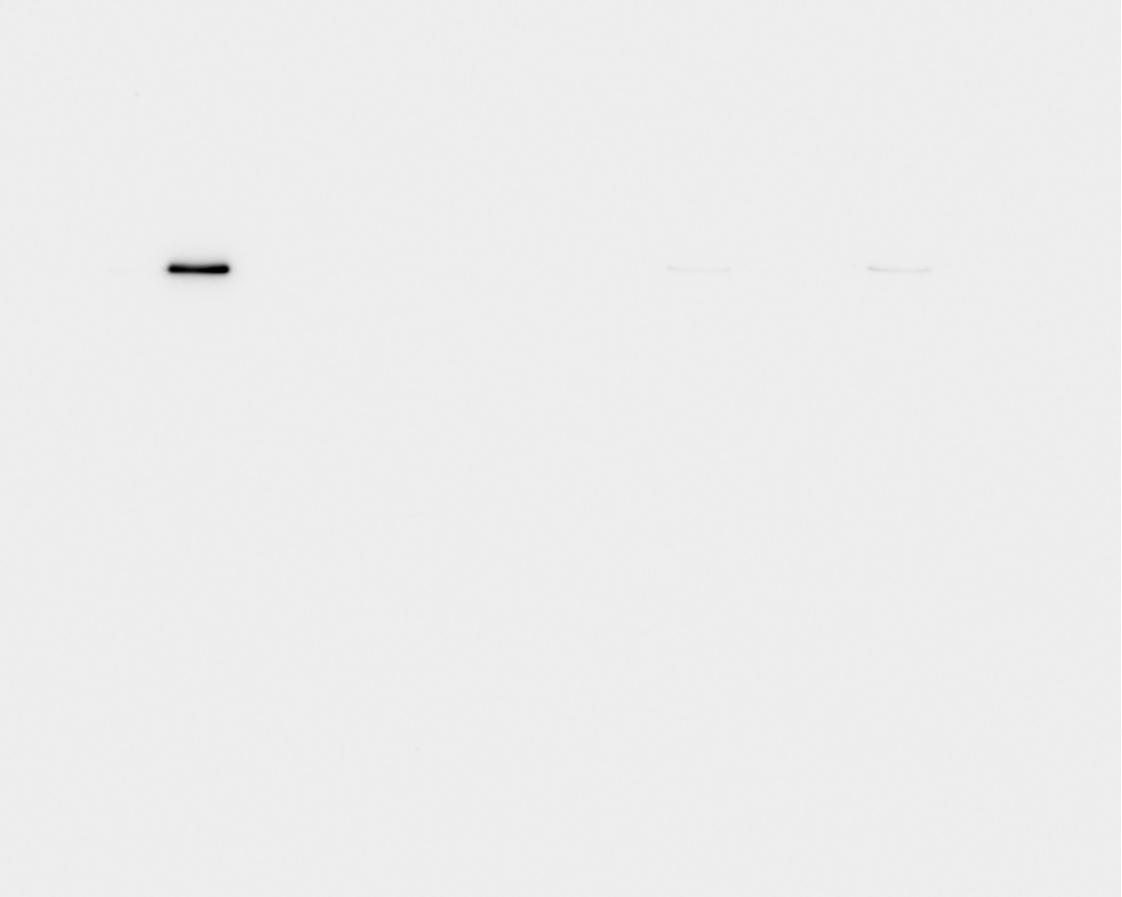

Supplement: Figure 5—figure supplement 2—source data 1. [file elife-74535-fig5-figsupp2-data1.zip › CK2-IncV_eLife3_Fig5S4_SourceData3.tif]

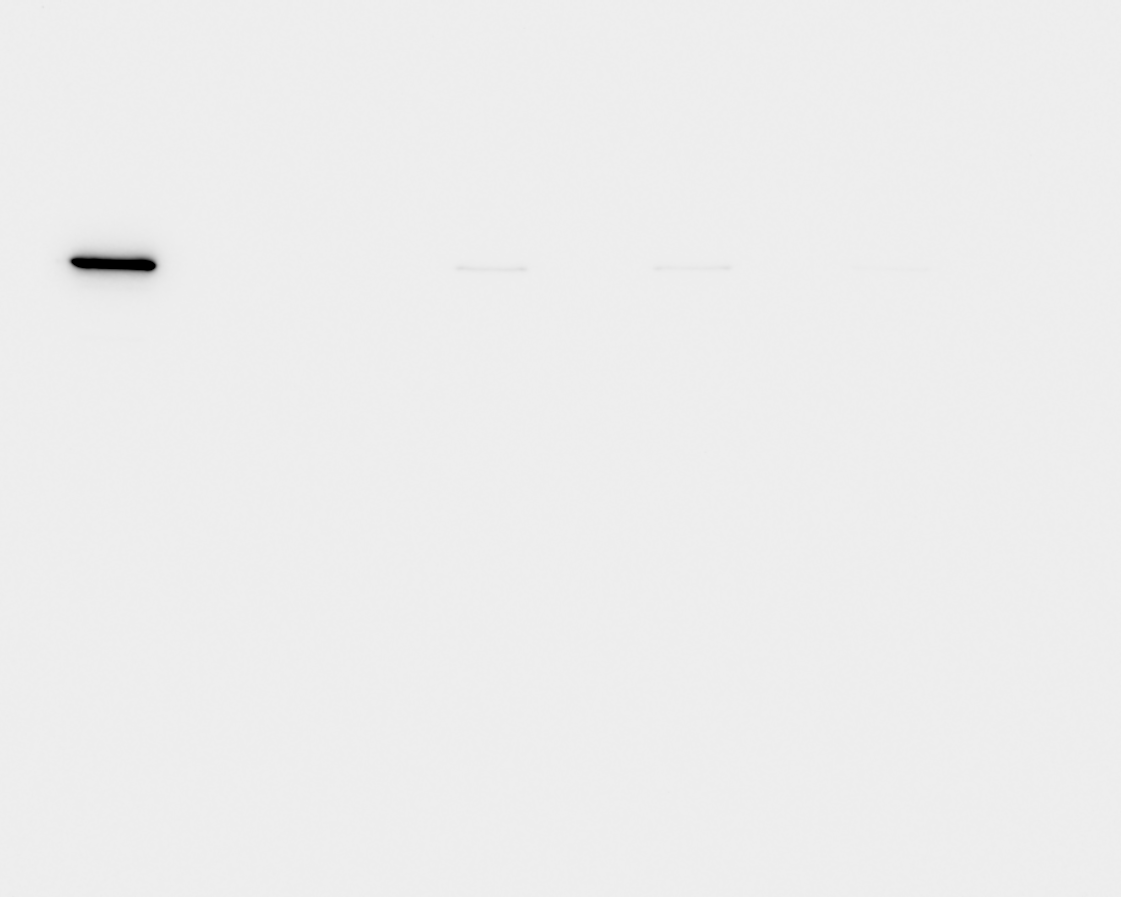

Supplement: Figure 6—figure supplement 2—source data 3. [file elife-74535-fig6-figsupp2-data3.tif]

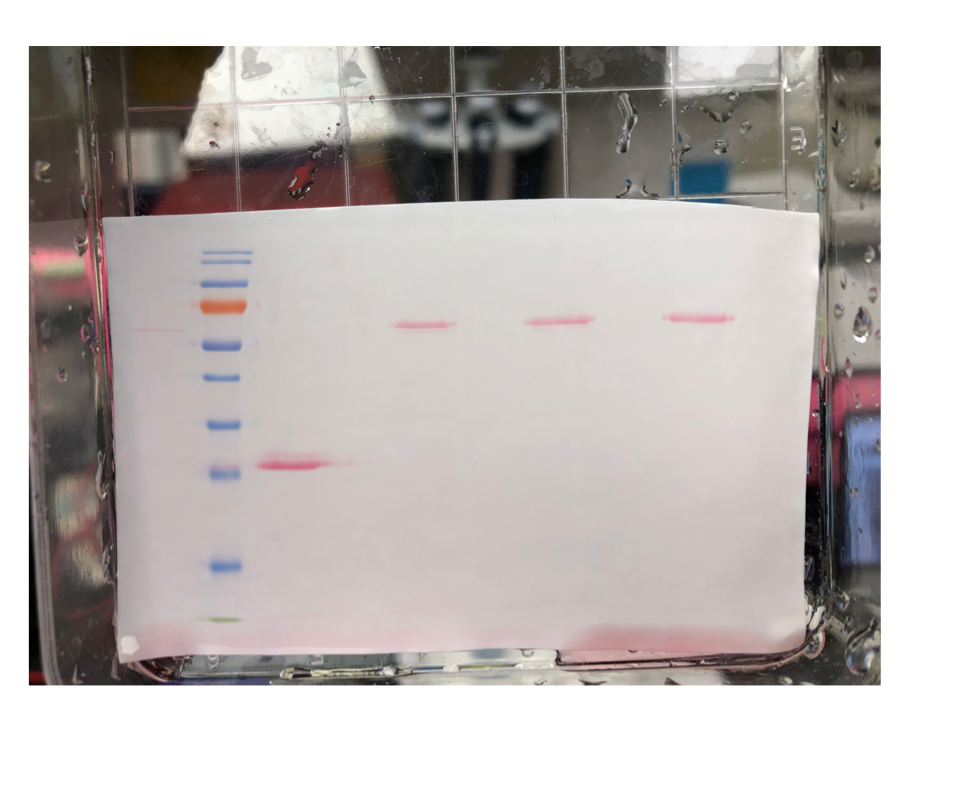

Supplement: Figure 6—figure supplement 2—source data 4. [file elife-74535-fig6-figsupp2-data4.tif]

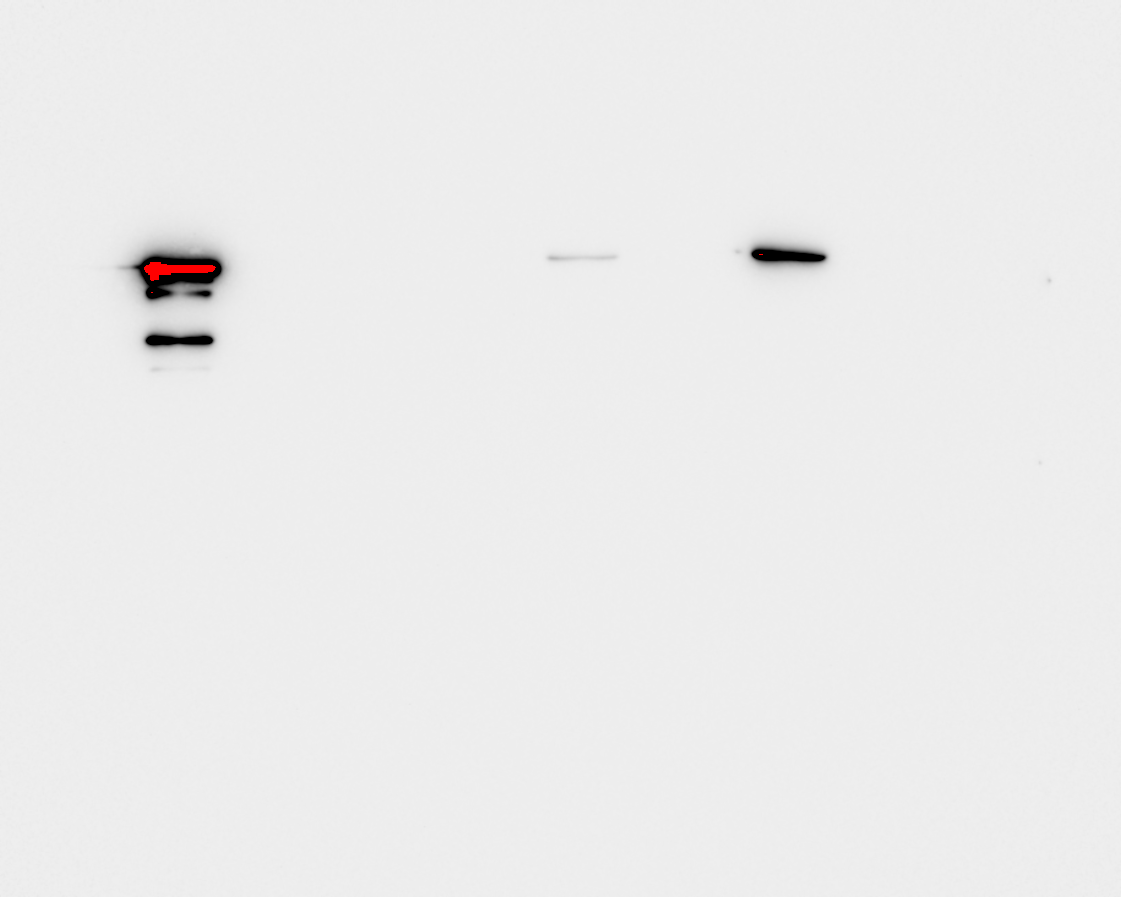

Supplement: Figure 7—source data 3. [file elife-74535-fig7-data3.tif]

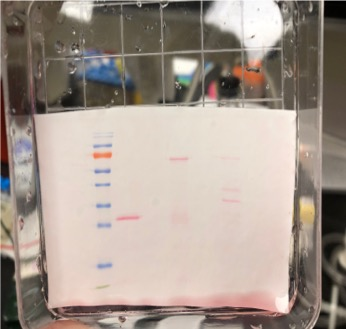

Supplement: Figure 7—source data 4. [file elife-74535-fig7-data4.tif]
